# Allergic Bronchopulmonary Aspergillosis (ABPA) Diagnosis Missed in the Context of Asthma Exacerbation Due to Medication Nonadherence

**DOI:** 10.7759/cureus.28202

**Published:** 2022-08-20

**Authors:** Waqas Rasheed, Saria Tasnim, Anass Dweik, Ola Al-Jabory, Stephen Usala

**Affiliations:** 1 Internal Medicine, Texas Tech University Health Sciences Center, Amarillo, USA

**Keywords:** asthma, allergic bronchopulmonary aspergillosis, uncontrolled asthma, aspergillus fumigatus, asthma exacerbations, abpa

## Abstract

Allergic bronchopulmonary aspergillosis (ABPA) results from a hypersensitivity reaction to *Aspergillus fumigatus* colonization of airways in patients with asthma or cystic fibrosis. Our patient is a 47-year-old female with a history of asthma and nonadherence to medications who presented with frequent asthma exacerbations. She required intubation three times within six months, labeled as asthma exacerbation due to nonadherence to medications until she was finally diagnosed with and successfully treated for ABPA. She was tested for ABPA very late as the medication nonadherence was thought to be the sole cause of repeated asthma exacerbations during previous hospitalizations. This case illustrates the importance of maintaining a high index of suspicion for ABPA in recurrent asthma exacerbation even in the setting of medical nonadherence.

## Introduction

Allergic bronchopulmonary aspergillosis (ABPA) is a rare disease and the diagnosis requires a high index of suspicion [1,2]. There are few respiratory fungal infections that can affect immunocompetent patients, *Aspergillus* being one of them. ABPA is a complex hypersensitivity reaction toward *Aspergillus fumigatus* presenting as difficult to control asthma with recurrent exacerbations. There are other fungal infections that can lead to a similar presentation; however, conventionally, ABPA is the term used when this is caused by *A. fumigatus*, and it is called allergic bronchopulmonary mycosis (ABPM) when the causative agent is a fungus other than *A. fumigatus* [2]. It increases morbidity and probably mortality if left untreated. We present a case of recurrent asthma exacerbation in a patient with a history of nonadherence to medication and active smoking, where the diagnosis of ABPA was delayed because of the history of medication nonadherence. The purpose of this case report is to highlight a frequent scenario where the diagnosis of ABPA can be missed or delayed.

The abstract of this case report has been published in Southern Regional Meeting 2022.

## Case presentation

A 47-year-old female with a history of asthma and nonadherence to medication presented to our hospital multiple times for dyspnea from asthma exacerbation, which was thought secondary to nonadherence to home medications including fluticasone furoate, umeclidinium, and vilanterol inhaler. She was also an active smoker. She was recently admitted for asthma exacerbation and treated successfully with intravenous steroids. The patient had been admitted six times in the past three years for dyspnea and was intubated every time during the most recent four hospital admissions. The hospital courses were similar: presentation with respiratory failure, the requirement for mechanical ventilation, and good response to standard breathing treatment and steroids followed by successful extubation and discharge. During the most recent hospitalization, she developed gradual onset exertional dyspnea, cough, and wheezing for a few days, which progressively got worse. She was intubated at home for hypoxic respiratory failure before being transferred to the hospital by emergency medical services. Laboratory workup showed hemoglobin of 12.3 gm/dL (12-16 gm/dL), white blood cells count of 11.2 x 10^9^/L (4-10 x 10^9^/L) with neutrophilic predominance, bicarbonate (HCO3) of 28 mEq/L (21-32 mEq/L), and arterial blood gases (ABGs) showed hypoxic and hypercapnic respiratory failure. *A. fumigatus* immunoglobulin E (IgE), total IgE, and total eosinophil count results are shown in Table 1.

**Table 1:** Laboratory findings IgE: immunoglobulin E.

Lab test	Result	Reference
*A. fumigatus* IgE	0.93 kU/L	0.35 kU/L
Total IgE	1804 IU/mL	1000 IU/mL
Total eosinophil count	>500 cells/microL	500 cells/microL

**Figure 1:**
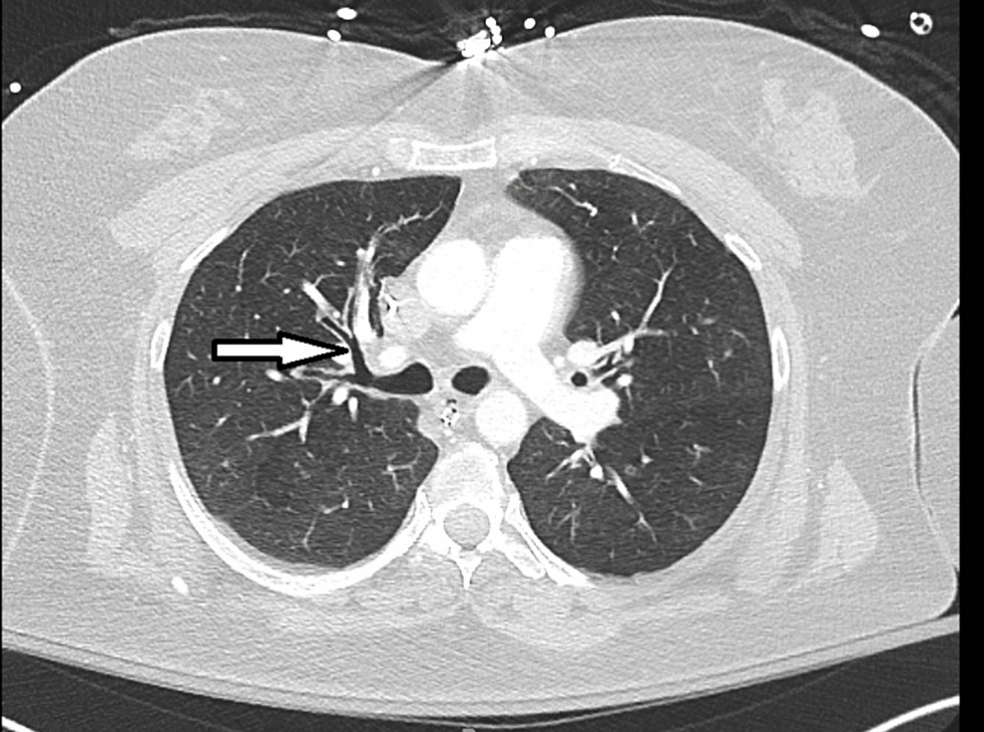
Dilated central airways seen in allergic bronchopulmonary aspergillosis (central bronchiectasis, arrow)

On physical examination in the ED, the vital signs were within normal limits, the patient was sleepy due to sedation, endotracheal tube in place, and the patient was on a ventilator, wheezing was noted on auscultation, and the rest of the physical exam was unremarkable. X-ray and CT scan of the chest showed clear lung fields with central bronchiectasis (Figure 1).

An echocardiogram showed normal left ventricular ejection fraction with possible mild diastolic dysfunction, and without any valvular disease. After she was successfully treated with intravenous steroids and extubated, the diagnosis of ABPA was suspected when the patient endorsed being compliant with medications for the last few weeks and still developing asthma exacerbation. The diagnosis was made from the diagnostic criteria of the International Society for Human and Animal Mycology (ISHAM) [3]. Following the diagnosis, she was started on itraconazole 200 mg twice daily for four months and prednisone 40 mg daily for three months resulting in improvement in symptoms and resolution of frequent hospital admissions for a follow-up period of several months after discharge.

## Discussion

ABPA is a rare disease and diagnosis; therefore, it requires a high index of suspicion. It should be considered with worsening respiratory symptoms in patients with underlying asthma or cystic fibrosis [1,2]. It presents as uncontrolled asthma, expectoration of brownish mucous, and hemoptysis [4]. Therefore, it can be misdiagnosed in patients having other risk factors for asthma exacerbation including nonadherence with medications, as seen in our patient. Central bronchiectasis is an important radiological feature of ABPA, as *A. fumigatus* most commonly affects the central airways (as shown in Figure 1) [5].

ABPA is a type-I, type-III, and possibly also a type-IV hypersensitivity reaction. *A. fumigatus* skin test is an important screening test and an important part of the diagnostic criteria. A positive test represents the presence of *A. fumigatus*-specific IgE antibodies [4-6]. Rarely, similar symptoms are caused by fungi other than *A. fumigatus*, known as ABPM, and the *A. fumigatus* skin test may be negative.

There are no single agreed-upon diagnostic criteria. We used the diagnostic criteria by the ISHAM for the patient (Table 2) [3].

**Table 2:** ABPA diagnostic criteria by the International Society for Human and Animal Mycology (ISHAM) Adapted from [3]. ABPA: allergic bronchopulmonary aspergillosis; IgE: immunoglobulin E.

Predisposing conditions (one required)
Asthma
Cystic fibrosis
Obligatory criteria (both required)
*A. fumigatus-*specific IgE > 0.35 kU/L or *A. fumigatus* skin test positive
Elevated total IgE > 1000 IU/mL, <1000 IU/mL acceptable if meets all other criteria
Other criteria (2/3 required)
*A. fumigatus-*specific IgG > 27 mg/L
Pulmonary opacities consistent with ABPA
Total eosinophils > 500 cells/microL in glucocorticoid naïve patient

Our patient was asthmatic, had elevated *A. fumigatus*-specific and total IgE, central bronchiectasis on CT scan chest, and elevated total eosinophil count on prior admissions before steroid treatment. There was no eosinophilia during the most recent admission as she had received steroids; however, eosinophilia was present during previous presentations (Table 1).

There are various reasons for misdiagnosis including atypical presentation and radiological findings, incorrect use and interpretation of screening tools, and the presence of pulmonary aspergillus overlap syndrome [6]. Pulmonary infiltrates can be misdiagnosed as pneumonia [7]. Medication nonadherence can be another important risk factor for failure of ABPA diagnosis, which is not usually recognized as a risk factor.

## Conclusions

We report a case of recurrent asthma exacerbation in the context of active smoking and medication nonadherence. There are many causes of asthma exacerbation including smoking, pollen, air pollution, respiratory infections, and medication nonadherence. Medication nonadherence can cause recurrent asthma exacerbations and it can keep a physician from thinking of ABPA, which may be an underlying contributing factor toward the recurrent asthma exacerbations, and the diagnosis may be missed or delayed in such cases. We emphasized the importance of maintaining a high index of suspicion for ABPA in patients with recurrent asthma exacerbation even if an alternate explanation for asthma exacerbation is present. Treating the underlying ABPA may improve or resolve recurrent asthma exacerbations, as observed in our case.

## References

[1] Zander DS (2005). Allergic bronchopulmonary aspergillosis: an overview. Arch Pathol Lab Med.

[2] Agarwal R (2009). Allergic bronchopulmonary aspergillosis. Chest.

[3] Agarwal R, Sehgal IS, Dhooria S, Aggarwal AN (2016). Developments in the diagnosis and treatment of allergic bronchopulmonary aspergillosis. Expert Rev Respir Med.

[4] Agarwal R, Chakrabarti A, Shah A (2013). Allergic bronchopulmonary aspergillosis: review of literature and proposal of new diagnostic and classification criteria. Clin Exp Allergy.

[5] Buckingham SJ, Hansell DM (2003). Aspergillus in the lung: diverse and coincident forms. Eur Radiol.

[6] Zou MF, Li S, Yang Y, Cao LL, Pan Y, Sun EH, Dong L (2019). Clinical features and reasons for missed diagnosis of allergic bronchopulmonary aspergillosis. (Article in Chinese). Zhonghua Yi Xue Za Zhi.

[7] Jiang N, Xiang L (2020). Allergic bronchopulmonary aspergillosis misdiagnosed as recurrent pneumonia. Asia Pac Allergy.

